# Optimization of Milk Substitutes for the Artificial Rearing of Chinese Tree Shrews (*Tupaia belangeri chinensis*)

**DOI:** 10.3390/ani12131655

**Published:** 2022-06-28

**Authors:** Jia-Qi Chen, Qingyu Zhang, Dandan Yu, Rui Bi, Yuhua Ma, Yijiang Li, Long-Bao Lv, Yong-Gang Yao

**Affiliations:** 1 National Resource Center for Non-Human Primates, National Research Facility for Phenotypic & Genetic Analysis of Model Animals (Primate Facility), Kunming Institute of Zoology, Chinese Academy of Sciences, Kunming 650107, China; chenjiaqi@mail.kiz.ac.cn (J.-Q.C.); zhangqingyu@mail.kiz.ac.cn (Q.Z.); yudandan@mail.kiz.ac.cn (D.Y.); birui@mail.kiz.ac.cn (R.B.); mayuhua@mail.kiz.ac.cn (Y.M.); liyijiang@mail.kiz.ac.cn (Y.L.); 2 Key Laboratory of Animal Models and Human Disease Mechanisms of the Chinese Academy of Sciences and Yunnan Province, and KIZ-CUHK Joint Laboratory of Bioresources and Molecular Research in Common Diseases, Kunming Institute of Zoology, Chinese Academy of Sciences, Kunming 650204, China; 3 Kunming College of Life Science, University of Chinese Academy of Sciences, Kunming 650204, China

**Keywords:** artificial rearing, inbred line, milk substitute, tree shrew, survival

## Abstract

**Simple Summary:**

The Chinese tree shrew, a squirrel-like mammal, has been widely used as a laboratory animal in biological research. However, the low survival rate of the pups has seriously hindered the establishment of inbred lines of this species and further limited its wider use. We found a milk substitute appropriate for artificial rearing of Chinese tree shrew pups independent of any obvious adverse effects on their survival, health, and reproductive performance compared to those of the maternally reared pups. The successful optimization of a milk substitute for the artificial rearing of Chinese tree shrew pups may increase the availability of this experimental animal.

**Abstract:**

The Chinese tree shrew (*Tupaia belangeri chinensis*) has the potential to replace the use of non-human primates in biomedical research. To increase the availability of this species, we have undertaken the ambitious task of establishing inbred lines of the Chinese tree shrew; however, we have been hindered by a low survival rate of inbred pups. Here, we report our artificial rearing (AR) of Chinese tree shrew pups using four different milk substitutes: the formula described by Tsang and Collins (milk TC) and three commercially available milk substitutes intended for possums (milk A and milk C) and for guinea pigs (milk B). We compared the effects of these milk substitutes and maternal milk on the daily milk consumption, growth performance, and survival of the pups. We also assessed the life span and reproductive performance of the F_1_ individuals given the best milk substitute as compared to the maternally reared (MR) pups. Milk B was found to be appropriate for AR. Pups fed with milk B had a high survival rate at the weaning age compared to those fed with the other milk substitutes. The AR pups fed with milk B had a life span similar to that of MR pups. AR females fed with milk B had an earlier age of the first reproduction, a larger number of litters, and a higher rate of survival of the offspring at the weaning age compared with the MR females. The successful optimization of a milk substitute for AR of Chinese tree shrew pups will undoubtedly facilitate the wide usage of this experimental animal.

## 1. Introduction

The Chinese tree shrew (*Tupaia belangeri chinensis*) is a small squirrel-like mammal. It is widely distributed throughout South China, Southeast and South Asia. There are morphological and biological variations [1,2] among the tree shrews from different regions, and a total of 15 species have been described in the genus *Tupaia* [3]. The Chinese tree shrew has a small body size (100–150 g), a short reproduction cycle (around 6 weeks from the time of conception to birth), and a low maintenance cost, which are all advantageous for its use as a laboratory animal [4]. Most importantly, the Chinese tree shrew has been found to be genetically close to primates [4,5,6,7].

In the past, researchers across the world have attempted to establish tree shrew models of hepatitis B virus and hepatitis C virus infections [8,9,10,11], SARS-CoV-2 infection [12], Kaposi’s sarcoma-associated herpesvirus infection [13], influenza viruses [14,15], and dengue virus [16] infections. Tree shrews were also used to create animal models of depression [17], osteonecrosis [18], and cancer [19,20]. Studies on tree shrews have also offered new insights into the fundamental mechanism of development of the visual system and brain function [21], as well as social avoidance and cooperative behavior [22,23].

For the past several years, we have been attempting to promote the usage of the Chinese tree shrew. Thus far, we have generated high-quality genome information [6,7], developed a successful transgenic technique using spermatogonial stem cells [24], and characterized several unique genetic features of the tree shrew immune system [25,26,27,28]. We also established two stable cell lines for tree shrews [29,30]. However, there are two main limitations to the further use of the tree shrew.

The first is the lack of resource sharing on how to manage this animal in captivity, and the second is the deficiency of related research reagents and behavioral testing models [4]. Although we have been undertaking the ambitious task of establishing inbred lines of the Chinese tree shrew since 2011, we have not achieved full success because most mothers consistently exhibit some of the forms of reproductive dysfunction, as is frequently seen in the domestication and inbreeding of wild animals [31,32,33,34,35,36].

Artificial rearing (AR) is used to improve the reproductive performance of various animal species in the laboratory [37]. It is well understood that the formulation of the milk substitute is particularly crucial to the survival, growth, and development of pups when employing hand-rearing methods. However, the maternal milk of the Chinese tree shrew is unique as it contains a high level of fat and protein, and a low level of lactose [38,39]. There have been several reports produced in the last few years that describe the use of milk substitutes for the AR of tree shrew pups. 

Pure bovine milk and baby formula milk with or without nutritional fortifiers have been proven to be inadequate. Tsang and Collins [40] invented a relatively successful milk substitute formula, which achieved a reasonably high survival rate (86%, 24/28) in neonatal tree shrews from Thailand. The validity and efficiency of this milk substitute were partially confirmed by Li et al. [41] and Liang et al. [42], although Liang et al. [42] observed a reduced weight gain of the Chinese tree shrew pups fed with a similar formula compared to the pups using the combination of maternal rearing (MR) and AR.

Here, we report our efforts at finding the best milk substitute for the pups of the Chinese tree shrews. We compared the effects of four milk substitutes with different nutritional compositions on the consumption of liquid food, growth performance, and the survival of tree shrew pups relative to those of MR pups. We also assessed the long-term effects of AR on life span, age at the first reproduction, litter size, number of litters, and survival rate of offspring at the weaning age of tree shrews fed with the best milk substitute compared to the MR pups. We found that milk B was the most successful milk substitute and can be recommended for the AR of Chinese tree shrew pups. This milk substitute may accelerate the establishment of inbred strains and further widen the use of this experimental animal.

## 2. Materials and Methods

### 2.1. Animals and Experimental Strategies

**Figure 1:**
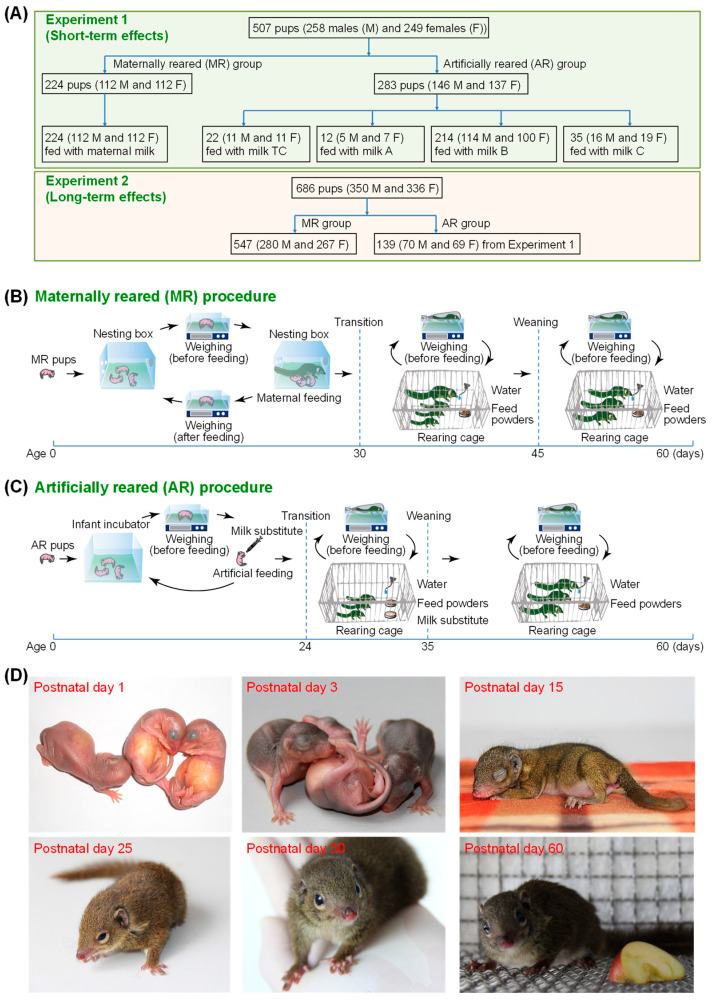
Experimental design and milk feeding procedures in this study. (**A**) Two experiments were conducted to evaluate the short-term effects (Experiment 1) and the long-term effects (Experiment 2) of milk substitutes on the survival and reproductive performance of the Chinese tree shrews. (**B**,**C**) Procedures of milk feeding for the tree shrew pups fed with maternal milk (**B**) and artificial milks (**C**) in Experiment 1. A total of 224 pups were included in the maternally reared (MR) group, and 283 pups were included in the artificially reared (AR) groups (milk TC, *n* = 22; milk A, *n* = 12; milk B, *n* = 214; and milk C, *n* = 35). Objects in (**B**,**C**) are not drawn to scale. (**D**) Photographs of the pups fed with milk B at different ages. On postnatal day 1, pups had smooth, pink skin with eyes closed, and their stomachs are full of milk. On postnatal day 3, pups had black skin due to pigmentation. On postnatal day 15, pups developed a white underbelly and a coat of brownish thick fur. On postnatal day 25, pups were able to move and have their eyes open. On postnatal day 30, pups became active and curious about their surroundings. On postnatal day 60, pups had a similar size and appearance to adults.

All tree shrews were obtained from the breeding colony of the experimental animal center of Kunming Institute of Zoology (KIZ), Chinese Academy of Sciences (CAS) during the breeding seasons of 2013–2021. Two experiments were conducted (Figure 1A).

In Experiment 1, which aimed to find the best milk substitute for the tree shrew pups. 224 pups (112 females and 112 males) from mothers with normal maternal behaviors were placed in the MR group; albeit the pups in this group were only allowed to be with their mothers for daily feeding sessions during the first 30 days. Pups from mothers with a history of bad maternal care and/or infanticide were randomly assigned to one of the four AR groups according to the order of birth. Ideally, the milk used for AR should start with a milk substitute that is as close as possible to the composition of the original breast milk of this species. 

However, we had difficulty collecting sufficient breast milk from mother tree shrews because only very small amounts of milk (usually less than 200 μL) can be sampled by hand. Therefore, we started AR with a formulation previously reported to be successful and commercially available formulations, even though these formulations differ substantially in composition from the original breast milk of the tree shrew, which is characterized by a high fat content [38,39]. 

We used four milk substitutes for AR: the Tsang and Collins formula (milk TC) [40] and three commercially available milk substitutes intended for pets (possums: milk A (possum milk substitute < 0.8, Wombaroo Food Products, Australia) and milk C (possum milk substitute > 0.8, Wombaroo Food Products, Australia); guinea pigs: milk B (guinea pig milk substitute, Wombaroo Food Products, Australia)). Soon after the experiment started, we found that compared with pups fed with milk B, more pups fed with milk TC, milk A, or milk C met the humane criteria for stopping the experiment, e.g., complete anorexia for up to 24 h, inability to obtain food and water, infections with no response to antibiotic therapy, marked change in behavior, signs of severe organ system dysfunction, and moribund state. 

For the sake of ethical considerations, we therefore adjusted the recruitment of pups into each AR group based on the effect of the respective milk substitute on the survival of pups. This is the reason for the markedly different sample sizes (milk TC: *n* = 22, including 11 males; milk A: *n* = 12, including 5 males; milk B: *n* = 214, including 114 males; and milk C: *n* = 35, including 16 males) of the four AR groups under comparison. We admit that the large difference in the sample size of each AR group might cause a bias in the evaluation of the effect of milks A and C on growth and survival. 

The parent tree shrews used in this experiment were genetically unrelated. After the completion of Experiment 1, the mothers (F_0_ generation) and 60-day-old tree shrews (F_1_ generation) were used in the project for establishing inbred lines of the Chinese tree shrews or other experiments. In total, 70 males and 69 females fed with milk B were returned back to the inbreeding colony, while others fed with milk B (25 males and 17 females), all pups fed with milk C (10 males and 8 females) and all pups in the MR group (86 males and 89 females) were used in other experiments.

In Experiment 2, we aimed to evaluate the long-term effects of the best milk substitute on the growth and development of AR tree shrews. The maintenance of these AR and MR tree shrews of the F_1_ generation in inbreeding colonies offered us an opportunity to discern potential long-term damaging effects of the best milk substitute on their growth and development. We performed a longitudinal observation based on the living 60-day-old tree shrews (F_1_ generation) fed with the best milk substitute from Experiment 1 together with some newly recruited 60-day-old tree shrews fed with maternal milk (F_1_ generation) in the inbreeding population. In total, 139 AR tree shrews (70 males and 69 females) fed with milk B and 547 MR tree shrews (280 males and 267 females) were included in this experiment.

### 2.2. Maintenance Procedures

In Experiment 1, mother tree shrews were housed individually in stainless steel cages (600 × 600 × 800 mm) with wooden nesting boxes (350 × 200 × 186 mm) on the back of the steel cages. The room temperature, light, and humidity were maintained at 24 ± 4 °C, 12-h dark/light cycle, and 55 ± 15%, respectively. The animals had *ad libitum* access to water and feed powders (including 22.2% crude protein, 4.9% crude fat, 1.2% crude fiber, 6.5% crude ash, 1.22% calcium, and 0.97% phosphorus, on a dry matter basis).

All pups were separated from their mother immediately after birth to avoid potential maternal infanticide. During the first 30 days after birth, the MR pups were wrapped in towels and kept in the nesting boxes (one to four pups per box). They were separated from their mothers except for the suckling time every morning (about 15 min, at 8:00 a.m.) based on the milk yields of the mother tree shrews and the observed feeding frequency (once a day or once per two days) of mother tree shrews in the previous studies [38,43]. 

Around thirty days later, the separation of the pups and mothers was ended, and the young, housed in the cages with their mothers, were allowed free access to breast milk, water, and feed powders. When the pups reached an approximate age of 45 days, their mothers were removed from the cages for weaning (Figure 1B). The AR pups, aged 1 day to 24 days, were wrapped in towels and reared in plastic boxes (350 mm × 200 mm × 186 mm; one to four pups per box), and placed inside an infant incubator (YP-90, Ningbo David Medical Device Co., Ltd., Ningbo, China). 

The temperature in the incubator was set at 32 °C and was gradually decreased by 0.5 °C every other day until to a final maintenance temperature at 24 ± 4 °C. The AR pups were orally fed with milk TC, milk A, milk B, or milk C (pre-warmed in 40 °C water) twice a day (at 8:00 a.m. and 16:00 p.m.) using sterile syringes (Figure 1C). The feeding frequency (twice per day) for each AR pup was determined on the basis of daily consumption of milk substitute and its gastric volume. Briefly, the daily consumption of each milk substitute was adjusted according to the weight and digestive capability of each AR pup. 

After around 24 days, the pups were transferred to stainless steel cages without their mothers (one to four pups per cage) and were maintained under a 12-h dark/light cycle at 24 ± 4 °C with a humidity of 55 ± 15% with free access to water and feed powders, which were the same as those offered to the mother tree shrews and the MR pups. A small amount of the respective milk substitute (1–2 mL) was placed in a dish in the cage to encourage self-feeding of the young. The volume of the respective milk substitute fed using the sterile syringe and dish was daily reduced until the weaning age of around 35 days (Figure 1C).

The maintenance procedure for tree shrews in Experiment 2 that were included in the inbreeding project was set as follows. Briefly, tree shrews with an age over 60 days were housed with their full siblings (one to two individuals per cage). After sexual maturity (around 4 months old), full siblings of different sexes were housed separately until mating (over 6 months old). The mating system was predominantly brother-sister inbreeding, with occasional parent-offspring inbreeding when there were no brother-sister pairs. When a female tree shrew was identified as pregnant, her male was transferred to another cage. 

The majority of the F_1_ pups were MR, except for those in the AR group of Experiment 1. However, most of mother tree shrews of the F_1_ generation and the subsequent generations had frequent infanticides no matter whether they were from the MR or AR groups, and the exact reason remained unknown. Therefore, we used AR for all the pups of the F_2_ generation and the subsequent generations that were born alive to prevent infanticide so that more inbred offspring could survive to adulthood. Milk B was chosen for AR of the inbred individuals based on the results of Experiment 1. The environmental conditions and feed powders for tree shrews with an age over 60 days were the same as those of the parent tree shrews in Experiment 1, and the maintenance procedure for inbred pups with an age under 60 days was the same as that of the MR or AR pups in Experiment 1.

### 2.3. Preparation and Chemical Analysis of Milk Substitutes

The milk TC (Tsang and Collins’ formula) was made from evaporated bovine milk (384 mL), pasteurized homogenized bovine milk (384 mL), two egg yolks without the yolk sac, and white Karo syrup (30 mL) following the previous report [40]. The milk A, milk B, and milk C were prepared according to the manufacturer’s instructions. The prepared milk substitutes were packed in 40 mL disposable sampling cups and stored in the freezer at −20 °C.

For chemical analysis of the artificial milks, the samples were transported to commercial testing companies (Pony Testing International Group Co., Ltd., Beijing, China and Qingdao Future Testing-Technology Co., Ltd., Qingdao, China) and were analyzed using the following methods as described in National Standards of China. Briefly, we followed the standard GB/Z 21922-2008 for measuring components related to energy and carbohydrate; GB 5009.8-2016 for measuring the amount of lactose; GB 5009.5-2016 for quantifying the protein contents, GB/T 5413.2-1997 for quantifying the amount of whey proteins and caseins; GB 5009.6-2016 for measuring the amount of fat; GB 5009.4-2016 for quantifying the amount of ash; GB/T 13885-2017 for measuring the concentrations of calcium, potassium, magnesium, iron, and zinc; GB/T 6437-2002 for measuring phosphorus; and GB 5009.268-2016 for quantifying the amount of sodium and copper. 

We used the following standards to quantify the amount of vitamin and other nutritional components: GB/T 17817-2010 for vitamin A, GB/T 14700-2018 3 for vitamin B_1_, GB/T 14701-2002 3 for vitamin B_2_, GB 5009.154-2016 for vitamin B_6_, GB 5413.14-2010 for vitamin B_12_, GB/T 17812-2008 for vitamin E, GB 5009.211-2014 for folic acid, GB 5009.259-2016 for biotin, GB/T 15400-1994 for tryptophan, and GB/T 18246-2000 for the other 17 amino acids.

### 2.4. Performance Evaluation

All pups were weighed individually using an electronic weighing scale (YP6102, Shanghai Guangzheng Medical Equipment Co., Ltd., Shanghai, China) before feeding every morning for the first 30 postnatal days and every 5 days at the morning time in the subsequent 30 days. Each pup was weighed directly without any anesthetic approach. If the animal was uncooperative, it was wrapped in a cloth bag before weighing; this was particularly needed as the pups grew larger. For pups in the MR group, animals were additionally weighed after feeding every morning in the first 30 postnatal days. The growth rate for pups was measured as total increase in body weight over a given period of time and was calculated according to the formula [44] as follow:

$$\mathrm{Growth}\;\mathrm{rate}=\left(W_{2}-W_{1}\right)/\left(T_{2}-T_{1}\right)$$

where *W*_1_ is the body weight of pup at time 1 (*T*_1_), and *W*_2_ is the body weight of pup at time 2 (*T*_2_).

The daily consumption of maternal milk of the MR pups during the first postnatal 30 days was measured as the difference between the daily body weight before and after suckling. The daily milk consumption of the AR pups during the first 24 postnatal days was measured by amount of milk fed by the syringe (1 mL, Chengdu XinjinShifeng Medical Apparatus & Instrument Co., Ltd., Chengdu, China) during feeding and was converted to weight data according to the density of each milk substitute (1.06 g/mL for milk TC, 1.06 g/mL for milk A, 1.09 g/mL for milk B, and 1.05 g/mL for milk C). The health condition and development of each pup were regularly checked by a veterinarian, and any observed abnormality was recorded. On humane grounds, sick animals were euthanized, and autopsies were performed to determine the cause of their deaths.

For all F_1_ individuals in the inbreeding project, the data about their life span, reproductive performance and health condition were recorded during 2013–2021. The life span of an individual was determined by subtracting the date of birth from the date of death. Individuals alive at the deadline for data collection (December 31, 2021) were excluded from the analysis of life span. 

Reproductive performance measurements included age at the first reproduction, litter size, number of litters, and survival rate of the offspring at the weaning age. Age at the first reproduction was the exact age of a tree shrew produced its first baby, and those animals without any offspring were excluded from the statistical analysis. Litter size was defined as the number of offspring produced at one birth by a parent tree shrew. We excluded these litters with dead fetuses or with offspring born alive but lost to infanticide immediately after birth from the analysis, to avoid distorting the litter size from the normal value. 

The number of litters was determined by counting the number of litters of offspring produced by a parent tree shrew, and individuals that produced no offspring were excluded from this analysis. The survival rate of the offspring at the weaning age was measured by the ratio of the offspring survived to the weaning age to the number of total offspring with exclusion of those born alive but lost to infanticide.

### 2.5. Statistical Analysis

We used the Spearman rank correlation test to investigate the potential relationship between daily milk consumption and the age of the pups, between body weight and the age of the pups, and between daily milk consumption and body weight of the pups. Differences in the daily milk consumption, body weight, and growth rate among different groups were evaluated by one-way analysis of variance (ANOVA), followed by post-hoc Bonferroni test. Welch ANOVA and post-hoc Tamhane’s T2 tests were used instead of ANOVA if the assumption of homogeneity of variance was violated. When the Shapiro–Wilk test for Gaussian distribution failed, the non-parametric Kruskal–Wallis test and post-hoc Dunn test were performed. 

On postnatal days 1 and 2, several pups that sucked colostrum immediately after birth were not measured for daily milk consumption and body weight, and were excluded from the analyses. Briefly, 203 pups on the first postnatal day and 44 pups on the second postnatal day in the MR group were excluded due to incomplete data for daily milk consumption and body weight. Several pups in the AR group on the first postnatal day (milk TC, *n* = 2; milk A, *n* = 4; milk B, *n* = 147; and milk C, *n* = 18) and the second postnatal day (milk A, *n* = 3; milk B, *n* = 89; and milk C, *n* = 10) were also excluded for the same reason. From postnatal day 3, complete data were collected for all living animals in both MR and AR groups. The 60-day survival function and survival rates at the weaning age were analyzed by the log-rank test and Fisher’s test, respectively.

**Figure 2:**
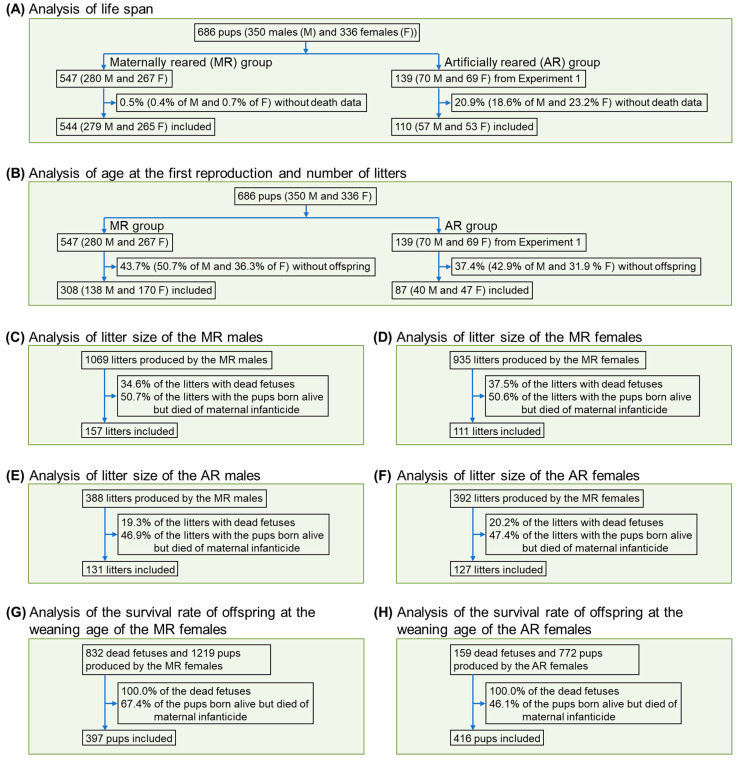
Enrollment of the F_1_ generation tree shrews in Experiment 2.

We estimated the life span and reproductive performance of the F_1_ generation tree shrews (*n* = 686) that met our selection criteria in the inbreeding colony. Briefly, we excluded the living animals before the deadline of data collection for analysis of life span (Figure 2A). For the analyses of age at the first reproduction and number of litters, we excluded the animals without production data (Figure 2B). For the analysis of litter size, we excluded the litters with dead fetuses, and the litters with offspring born alive but that died of infanticide on the day of birth (Figure 2C–F). 

For analysis of the survival rate of offspring at the weaning age, we excluded the dead fetuses and the pups born alive but that died of infanticide at the day of birth (Figure 2G,H). As the values of life span, age at the first reproduction, litter size, and number of litters failed to pass the Shapiro–Wilk test for normal distribution, we used the Mann–Whitney U test to determine the significance for each gender between MR and AR individuals. Since pups in the MR group were only raised by mother tree shrews, the survival rate of offspring at the weaning age between MR and AR groups was analyzed for the mother tree shrews using Fisher’s exact test.

The mean values of the daily milk consumption, body weight, life span, age at the first reproduction, litter size, and number of litters are presented as the mean ± standard error of the mean, whereas the survival rate of individuals and their offspring at the weaning age are given as a percentage. A *p* value < 0.05 was considered as statistically significant. Statistical analysis was performed by the Prism (v8.0.2.263, GraphPad Software, San Diego, CA, USA) using the tree shrew pup as the experimental unit.

## 3. Results

### 3.1. Nutritional Composition of Different Milk Substitutes

We performed a compositional analysis for all the milk substitutes, which showed varying proportions of protein (5.44%~8.23%), fat (2.40%~7.33%), and energy (304~478 kJ/100 g) on a wet matter basis (Table 1). The ratio of protein to fat was approximately close to 1:1 in milk TC (5.71:6.24) and milk C (8.23:7.33) but increased to an approximate ratio of 2:1 in milk A (5.44:2.40) and milk B (7.70:4.33). The ratios of caseins to whey proteins of milk A (1.50:3.37), milk B (2.50:4.36), and milk C (2.03:5.08) were much lower than that of milk TC (4.32:0.33). In general, milk A had a much lower content of most nutrients, including energy, crude fat, lactose and ash, compare with the other milk substitutes (Table 1). 

We compared the components of each milk substitute with the reported values of tree shrew maternal milk by Yang et al. [39]. All milk substitutes had a lower content of protein and crude fat than maternal milk and had a higher content of lactose than maternal milk (Table 1).

### 3.2. Daily Consumption of Liquid Diet before Introduction of Solid Food

In order to find an optimal milk substitute for the Chinese tree shrew pups, we conducted a feeding experiment based on a total of 507 pups. The animals were assigned into the MR group (*n* = 224; Figure 1B) and the AR group (*n* = 283), with the AR group being further divided into four sub-groups fed with milk TC (*n* = 22), milk A (*n* = 12), milk B (*n* = 214), and milk C (*n* = 35) (Figure 1C), respectively. A correlation between the daily milk consumption and the age of pups in each group and a comparison of the daily milk consumption among the groups was performed. 

**Figure 3:**
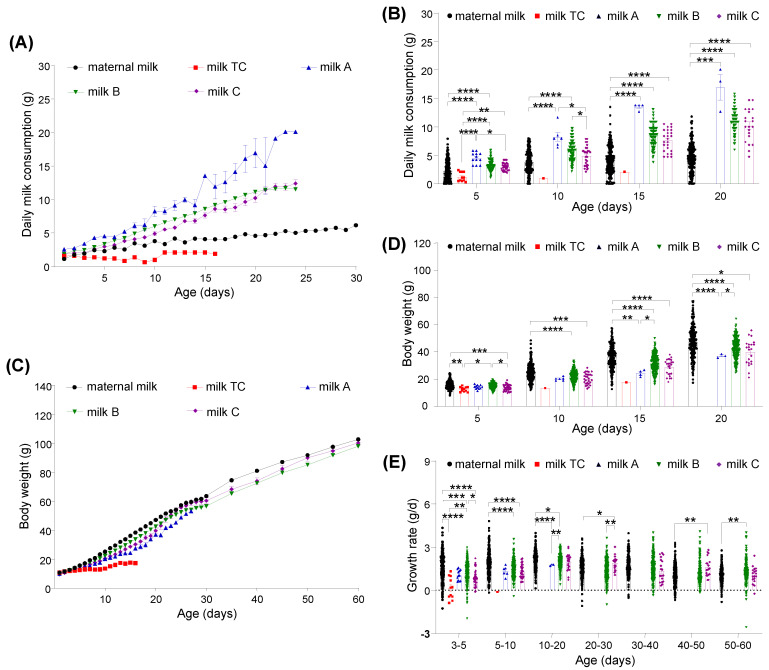
Daily milk consumption and growth of Chinese tree shrew pups fed with different types of milk. (**A**) Daily consumption of milk before the introduction of solid food powders. Maternal milk, *n* = 224; milk TC, *n* = 22; milk A, *n* = 12; milk B, *n* = 214; and milk C, *n* = 35. A total of 41 pups fed with maternal milk, all 22 pups fed with milk TC, 11 pups fed with milk A, 30 pups fed with milk B, and 16 pups fed with milk C died at different ages during the measurement period. (**B**) Daily milk consumption by pups on postnatal days 5, 10, 15, and 20. (**C**) Growth curve of the Chinese tree shrew pups. A total of 49 pups fed with maternal milk, all 34 pups fed with milk TC or milk A, 33 pups fed with milk B, and 17 pups fed with milk C died at different ages during the test. (**D**) Body growth of tree shrew pups on postnatal days 5, 10, 15, and 20. (**E**) Growth rate of the pups during postnatal days 3–5, 5–10, 10–20, 20–30, 30–40, 40–50, and 50–60. * *p* < 0.05, ** *p* < 0.01, *** *p* < 0.001, **** *p* < 0.0001. Values are presented as the mean ± SEM.

We found that the daily consumptions of maternal milk (r = 0.983, *p* < 0.0001), milk TC (r = 0.511, *p* = 0.046), milk A (r = 0.993, *p* < 0.0001), milk B (r = 0.997, *p* < 0.0001), and milk C (r = 0.998, *p* < 0.0001) were all positively correlated with the age of pups (Figure 3A), suggesting that these milk substitutes could roughly sustain the growth needs of the pups, especially milk B and milk C. Compared to the MR pups, pups fed with milk A, milk B, and milk C had a higher daily milk consumption while pups fed with milk TC had a lower consumption (Figure 3A). 

We further compared the pup daily milk consumption on postnatal days 5, 10, 15, and 20 among the four sub-groups in the AR group and the MR group, pups fed with milk A had a generally higher daily milk consumption than those pups fed with other milk substitutes (Figure 3B). Although the daily milk consumption might be biased by the syringe feeding and suckling, the longitudinal monitor of daily milk consumption reflected different bioavailability or amount of nutrient and energy to meet the tree shrew’s needs for growth that were supplied by different liquid foods.

### 3.3. Growth Performance of Pups Fed with Different Milks

The results from the correlation analysis between body weight and pup age in each group showed that the body weights of the MR pups (r = 1.000, *p* < 0.0001), of the pups fed with milk TC (r = 0.962, *p* < 0.0001), milk A (r = 0.999, *p* < 0.0001), milk B (r = 1.000, *p* < 0.0001), and milk C (r = 1.000, *p* < 0.0001) increased significantly along with their ages in each group (Figure 3C). However, comparison of the body weight and growth rate for each group of pups at different postnatal days indicated that each group of pups had different growth patterns. During postnatal days 1 and 2, there were no differences among nearly all groups of pups except for the pups fed with milk C, which had a significantly lower body weight (9.41 ± 0.23 g for postnatal day 1, 10.66 ± 0.26 g for postnatal day 2) than the MR pups (10.64 ± 0.32 g for postnatal day 1, 11.52 ± 0.11 g for postnatal day 2), the pups fed with milk TC (10.57 ± 0.23 g for postnatal day 1), and the pups fed with milk B (10.46 ± 0.14 g for postnatal day 1, 11.54 ± 0.12 g for postnatal day 2), respectively. 

On postnatal day 3, pups in different groups showed a similar body weight. Subsequently, the AR pups exhibited a slight growth delay in comparison with the MR pups, indicating that pups fed with artificial milk were subjected to early life undernutrition caused by the inadequacy in quality and/or quantity of the artificial milk. Pups fed with milk TC, milk A, milk B, and milk C had a smaller weight than those of the MR pups on most of feeding days (Figure 3C). Among all four groups of the AR pups, those fed with milk B had a higher body weight than the other pups in the AR groups. 

For instance, the pups fed with B milk weighed significantly more (*p* < 0.05) than the pups fed with milk TC or milk C on postnatal days 5 and the pups fed with milk A on postnatal days 15 and 20 (Figure 3D). When the pups fed with milk B and milk C started to take solid food, they grew quickly to catch up with the MR pups, even with a significantly higher (*p* < 0.05) growth rate than that of the MR pups (during postnatal days 40–50 for the pups fed with milk C and during postnatal days 50–60 for the pups fed with milk B) (Figure 3E), and the weight difference between the MR pups and the pups fed with milk B progressively narrowed during the growth period (Table 2). 

All these observations suggested that the unrestrained solid food had sufficient or better nutrition for growth, which also led to an earlier weaning age for pups fed with milk B and C than the pups in the MR group. The pups fed with milk TC or milk A did not show a similar growth pattern as observed in the pups fed with milk B and milk C, possibly because all pups fed with milk TC and the majority (91.7%, 11/12) of the pups fed with milk A died before the animals started to take solid food. In addition, the standard error of the mean of body weight in the AR groups was smaller than that of the MR group in most cases (Table 2), indicating that the AR groups have a lower variability in body weight than the MR group.

**Figure 4:**
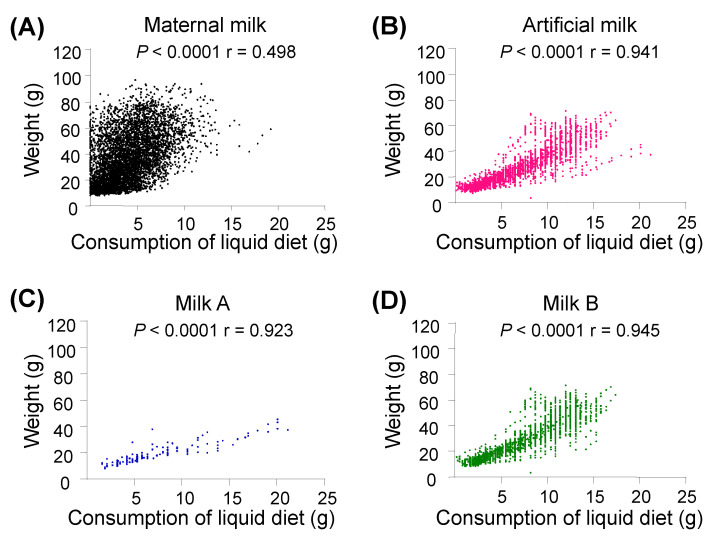

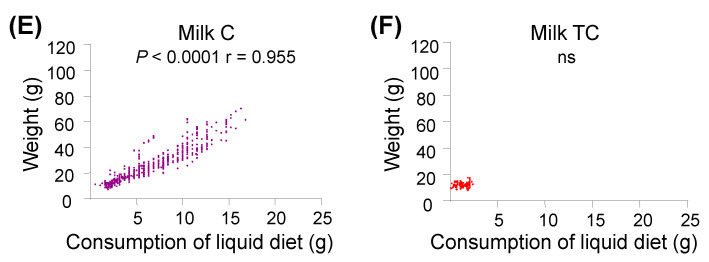
The correlation between body weight and daily milk consumption in the tree shrew pups fed with different milks. The correlation between body weight and daily milk consumption in pups (for pups fed with maternal milk (**A**), postnatal days 1–30; for pups fed with artificial milk (**B**), including milk A (**C**), milk B (**D**), milk C (**E**), and milk TC (**F**), postnatal days 1–24). A *p* value < 0.05 was considered as statistically significant; and ns, not significant.

We further performed a correlation analysis to show the increase of body weight and daily milk consumption. We found that the body weights of the MR pups (r = 0.498, *p* < 0.0001; Figure 4A), the pooled AR pups (r = 0.941, *p* < 0.0001; Figure 4B), and the AR sub-groups (Figure 4C–E) except for pups fed with milk TC (*p* = 0.101; Figure 4F), were positively correlated with their daily consumption of milk before introduction of solid food powders. The exact reason why milk TC did not prove as successful as described in the previous report [40] remains to be explored.

### 3.4. Survival of Pups Fed with Different Milks

We investigated the survival of the pups in each group fed with the different milk substitutes or breast milk. Kaplan–Meier survival analysis revealed that the feeding milk significantly affected the 60-day overall survival of the pups (*p* < 0.001). The pups fed with milk B had the best overall survival, with a slightly higher rate compared with that of the MR pups, followed by pups fed with milk C. 

**Figure 5:**
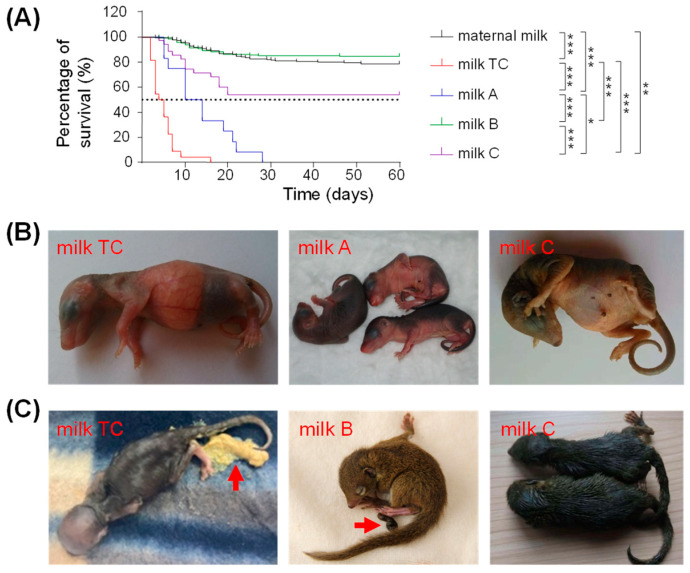
Survival curves and morphology of pups fed with maternal milk and artificial milk. (**A**) Kaplan–Meier survival curves of pups fed with different milks. Maternal milk, *n* = 224; milk TC, *n* = 22; milk A, *n* = 12; milk B, *n* = 214; and milk C, *n* = 35. * *p* < 0.05, ** *p*< 0.01, and *** *p* < 0.001. (**B**,**C**) Representative pictures of pups fed with different milk substitutes. (**B**) On postnatal day 4, pup fed with milk TC was skinny and has gastric food retention; pups fed with milk A had a completely empty stomach before feeding every morning; pups fed with milk C had mild diarrhea. (**C**) Pup fed with milk TC on postnatal day 13 had diarrhea with liquid, yellow stool (indicated by an arrow) and dehydration. Pup fed with milk B on postnatal day 22 had brown, soft, sausage-shaped feces (see arrow). Pups fed with milk C on postnatal day 18 had severe diarrhea.

Pups fed with milk A and milk TC had a worse overall survival (Figure 5A). All 22 pups fed with milk TC died before postnatal day 17 and all 12 pups fed with milk A died before postnatal day 29. In contrast, the majority of the MR pups (79.9%, 179/224) and pups fed with milk B (85.0%, 182/214) survived to the weaning age at postnatal day 45 and postnatal day 35, respectively. Pups fed with milk C (54.3%, 19/35) had a lower survival rate than pups fed with milk B although pups in both groups had a similar weaning age at postnatal day 35.

Autopsy of the dead pups showed that digestive disease was the leading cause of the death before the weaning age for all pups (Table 3). Nonetheless, the pups fed with milk TC seemed to be more vulnerable to digestive disease (90.9%, 20/22) than the dead pups in the MR group and three sub-groups of the AR group (47.6%, 50/105). The pups fed with milk TC frequently had severe gastric food retention (Figure 5B). A total of 20 out of 22 pups fed with milk TC had watery diarrhea with yellow stool (Figure 5C), and eventually died of dehydration within a few days. The pups fed with other types of milk had no such severe gastrointestinal problems (Figure 5B) and their excrement was usually in strip form with a brown color (Figure 5C). Although a few pups fed with milk A, milk B, and milk C had diarrhea, the symptoms were not as severe as those pups fed with milk TC.

### 3.5. Longitudinal Observation on the Feeding Effect of the Optimal Milk Substitute

We performed a longitudinal observation looking at the health and growth of the pups fed with milk B, to evaluate any unfavorable effects. Based on an observation that 179 of 214 pups (83.6%) fed with milk B survived to adulthood (aged 6 months or more), we found no cases with abnormal health conditions, except for a slight growth delay in the early days of life relative to the MR pups. A follow-up observation for these animals up to 7 years or more (there were 29 tree shrews alive to date as the others died or were used for experiments) showed no abnormalities. Neither did we find any cases of abnormal health conditions, based on an observation for 2329 individuals out of 3254 inbred pups of F_2_ generation and the subsequent generations fed with milk B and surviving to adulthood in our inbreeding project.

We retrieved all the death and reproduction records covering the period 2013–2021 for the F_1_ generation tree shrews (including the AR individuals fed with milk B in Experiment 1) in the inbreeding project, to investigate the potential effect of milk B feeding on the life span and reproductive performance of these animals. As shown in Table 4, no significant differences were observed for life span between tree shrews fed with breast milk and milk B (male, *p* = 0.712; female, *p* = 0.217). We observed no significant difference for litter size between tree shrews fed with breast milk and milk B (male, *p* = 0.885; female, *p* = 0.714), neither. 

However, AR female tree shrews had a significantly earlier age at the first reproduction (*p* < 0.001) and produced more litters of offspring (*p* < 0.001) than those MR female animals. The offspring produced by these AR female tree shrews had a significantly higher survival rate (*p* < 0.001) at the weaning age (85.8%, 357/416) than that of MR female tree shrews (74.1%, 294/397). Nonetheless, these data should be received with caution, as the reproductive opportunities of AR and MR female tree shrews and human influences on both groups might have some differences due to the inbreeding strategy. Moreover, the sample sizes of animals in the MR group and the AR group were different, which may also lead to a biased evaluation for the significance of certain parameters.

Taken together, it appears that milk B almost meets the nutritional needs of the Chinese tree shrew, and milk B can be used as a valid artificial milk for feeding pups when required.

## 4. Discussion

The Chinese tree shrew has many advantages as an experiment animal, especially considering the fact that it is genetically close to primates [5,6,7]. However, this animal has continued to be under-utilized because of the lack of access to resources, specific analytical reagents, and inbred lines [4]. In order to promote the wider use of the Chinese tree shrew, 10 years ago, we began a project to establish inbred lines of this experimental animal. However, we quickly encountered the obstacle that most inbred pups reared with their mothers failed to survive to their weaning age, simply because many mothers of these inbred pups declined to nurse their pups, and some mothers cannibalized the pups immediately after birth. Maternal infanticide has been observed in many animals, with different speculations underlying this abnormal behavior [45,46,47]. 

At present, the exact etiology of maternal infanticide in tree shrews is unclear. Given the fact that genetic, environmental [48], hormonal, and experiential factors [49] are involved in maternal infanticide, multiple possibilities for health-related issues may account for the high rate of tree shrew maternal infanticide, and this deserves focused study in the future. Apart from the maternal infanticide, we observed the effects of inbreeding depression and the occurrence of spontaneous diseases in the inbred populations; similar to the reports for other inbred animals [32,36,50]. We therefore needed to consider the use of AR, an effective approach for improving the survival of infant mammals, albeit time-consuming, to solve the problem of the loss of inbred pups of the Chinese tree shrews in our inbreeding project. 

Initially we started by using tree shrew breast milk; however, we had difficulty in collecting sufficient milk from mother tree shrews because of the very small amount of milk that could be obtained from each donor. Therefore, we needed to consider using milk substitutes, such as milk TC [40] and other milks sold for feeding pets, even though the formulations differed substantially in their components when compared to that of the breast milk of the tree shrew.

There have been several attempts at AR of tree shrew pups reported in previous studies [40,41,42], although the outcomes of AR with the same or a similar formula in these studies were not completely consistent [40,41,42]. In our study, we found that pups fed with milk TC and milk A had the poorest overall survival, with none surviving to their weaning age. There are two possibilities to explain these inconsistencies. First, the exact nutritional components and quality of the milk TC made by the original method [40] might be different from our milk TC, although we followed the same reported recipe. Second, the maintenance procedures used in this study were also slightly different from those used by Tsang and Collins [40]. 

We do not know whether our experiments were conducted under different conditions as compared to the previously reported protocol [40] and whether this would have affected the survival of pups fed with milk TC. It is unlikely that this performance difference was caused by potential differences of digestive physiological characteristics among different tree shrew subspecies/species, as milk B and milk C (which were best for feeding guinea pig and possum, respectively) were capable of hand rearing the Chinese tree shrew pups, albeit with different performance.

In order to find an optimal milk substitute for AR of Chinese tree shrew pups and to evaluate the long-term feeding effect, we compared the performance of four milk substitutes (milk A, milk B, milk C, and milk TC) with that of maternal milk. We were finally able to show that milk B was a relatively optimal milk substitute that provided acceptable growth and survival performance. More importantly, AR with milk B improved the reproductive performances (e.g., an earlier age at the first reproduction, a larger number of litters, and a higher survival rate of offspring at the weaning age) of female tree shrews. 

There are several possible reasons for the success of milk B, although there are still some limitations to be considered. First, milk B is made from ingredients digestible by newborn guinea pigs and does not contain any yolk protein. Whereas, in milk TC [40] yolk proteins are one of the dominant proteins. It has been shown that yolk proteins are poorly utilized by mammals, giving a low bioavailability due to a high proportion of phosvitins that are resistant to proteolytic action [51]. Second, milk B has a reduced ratio of caseins and whey protein compared to that of milk TC. 

Caseins are more difficult to place into solution, digest, and absorb compared with whey proteins [52,53], and this may be one possible reason leading to functional gastrointestinal disorders [54]. Third, although milk B has a relatively better nutrient profile compared to other milk substitutes, it has a relatively low fat content but a high protein content and is therefore not complete or balanced in terms of nutrient concentrations and ratios when compared to maternal milk. The concentration of fat in milk B (Table 1) is one-fifth of that reported in tree shrew breast milk [38,39]. 

As a result, pups have to consume excessive proteins to obtain sufficient energy for maintaining normal physiology and growth, and this may have long-term adverse effects on the metabolic system [55,56] and excretory system [57]. Therefore, it may still be possible to improve the formulation of milk B and more closely satisfy the nutritional requirements of tree shrew pups; although thus far, we have not observed any abnormal health conditions related to metabolic disorders. Milk C is very similar to milk B in its concentrations of most of the nutritional components except that it has a lower ratio of protein to fat; however, the survival of pups fed with milk C was lower than that of pups fed with milk B. This observation indicated that other factors, such as fatty acid composition and palatability, should be considered.

According to Yang et al. [39], the maternal milk of tree shrew contains a higher proportion of protein and fat than the bovine milk [58]. It is thus reasonable that the lactose content is very low in tree shrew maternal milk considering the fact that there is a negative correlation between the content of lactose and fat for the regulation of nutrient density in milk [59]. The high density of protein and fat in tree shrew maternal milk may be the reason for a rapid growth of the pups and a long intersucking interval [38,43]. It is unfortunate that we did not collect sufficient breast milk from mother tree shrews for quantification of the chemical components and compared to the milk substitutes in this study. A focused study of the major components and trace elements in tree shrew maternal milk by using very small amount of milk sample is warranted for better optimizing milk substitutes for tree shrew pups.

The milk substitute can be delivered to tree shrew pups by syringe feeding and bottle feeding. Syringe feeding has several advantages and disadvantages. First, it is a delivery method for accurate individual intake of liquid food or dosing of medications [60]. Second, it fits for feeding a tree shrew of any age. Third, feeding with disposable syringes may be less risky in terms of bacterial contamination resulting in diarrhea and infections than the bottle feeding [61]. 

However, this procedure is time-consuming and requires some patience and expertise to avoid tooth breaking and aspiration pneumonia in the pups. In contrast, bottle feeding is typically considered as a more natural method as it mimics the young animal suckling from the mother, yet it is useless when the pup is too young, sick or is refusing to eat or drink. We therefore suggest that the syringe feeding or bottle feeding should be used according to the healthy condition of the pups and practicability of the feeding tools.

An interesting finding is that there was a relatively lower variability in body weight in the AR group fed with milk B compared to the MR group. The control of inter-individual variation in body weight may benefit the “reduction” in 3Rs [62] (reduction of sample sizes when designing an animal experiment). The exact reasons for the uniformity in body weight in the AR group remain unknown. It is possible that the maternal effects, such as the milk yield and milk composition [63,64] in MR, were partly eliminated in AR, and the competitive abilities of pups for resources [65] in MR were normalized by control feeding in AR since the growth of pups heavily depends on their milk intake and the milk composition. Whether the rearing performance for MR of outbred and inbred lines would differ significantly is an interesting question to be tested in the future.

It is worth mentioning that the current study has some limitations on sample size and constraints of hand rearing, especially considering the fact that the sample size of animals in each AR group was different, which may cause a bias for estimating the performance of each milk substitute. We are not trying to encourage AR as this is not completely comparable to MR, and it may yet be shown to have a long-term effect on the development of animals, especially considering the effects of maternal separation [66,67]. Nevertheless, this approach should be considered when there is a need to increase the survival rate of the pups. 

The demands for more experimental animals as valid models for studying diverse human diseases and their unique characters for modeling have fueled the ongoing interest in the laboratory domestication of wild animals, despite the fact that many barriers have hindered their laboratory domestication [68]. Hopefully, our success in building and maintaining Chinese tree shrew breeding lines with the use, when appropriate, of the hand-rearing method with an appropriate milk substitute may provide some insight into how to overcome some of the difficulties, and set a good example of how to solve the problems that have previously limited the laboratory domestication of wild animals.

## 5. Conclusions

In conclusion, we found a relatively successful milk substitute for the Chinese tree shrew. Pups fed with this milk had a comparable survival rate to pups reared by their mothers and had no apparent abnormality of growth. The female tree shrews artificially reared with this milk had an earlier age of first reproduction, a larger number of litters, and an improved survival of offspring. We believe the current study might hasten the establishment of inbred lines of the Chinese tree shrew and extend the applications of this animal in various fields of biological and biomedical research. This study may also offer some help to researchers struggling with breeding issues in the domestication of other wild animal species for scientific purposes.

## Figures and Tables

**Table 1:** Chemical components of different milk substitutes and maternal milk.

Item	Milk TC ^(a)^	Milk A ^(b)^	Milk B ^(c)^	Milk C ^(d)^	Maternal Milk ^(e)^
Energy (kJ/100 g)	469	304	375	478	NA ^(f)^
Protein (g/100 g)	5.71	5.44	7.70	8.23	10.41 ± 0.82
whey proteins (g/100 g)	0.33	3.37	4.36	5.08	NA
Caseins (g/100 g)	4.32	1.50	2.50	2.03	NA
Crude fat (g/100 g)	6.24	2.40	4.33	7.33	26.01 ± 1.63
Carbohydrate (g/100 g)	8.00	6.24	4.47	6.55	NA
Lactose (g/100 g)	4.10	0.54	3.53	1.68	0.45 ± 0.03
Ash (g/100 g)	1.10	0.64	1.16	1.53	0.99 ± 0.61
**Minerals**					
Sodium (mg/100 g)	69.60	92.16	61.37	115.75	77.21 ± 11.88
Calcium (mg/100 g)	130.00	100.80	166.82	195.00	329.19 ± 75.87
Phosphorus (mg/100 g)	160.00	124.80	128.63	200.00	341.45 ± 24.50
Potassium (mg/100 g)	200.00	100.80	172.52	187.50	137.37 ± 28.47
Magnesium (mg/100 g)	23.00	12.48	17.75	23.25	25.62 ± 5.45
Iron (mg/100 g)	0.67	0.46	0.72	0.68	1.48 ± 0.45
Zinc (mg/100 g)	ND ^(g)^	ND	0.59	1.08	1.22 ± 0.16
Copper (μg/100 g)	ND	111.75	ND	105.50	620.38 ± 371.75
**Vitamins**					
Vitamin A (IU/100 g)	213	145	270	615	NA
Vitamin E (mg/100 g)	0.30	1.17	1.32	2.02	NA
Vitamin B_1_ (mg/100 g)	0.06	0.14	0.16	1.20	NA
Vitamin B_2_ (mg/100 g)	0.53	0.37	0.39	0.67	NA
Vitamin B_6_ (μg/100 g)	10.00	8.00	133.38	10.00	NA
Vitamin B_12_ (μg/100 g)	0.78	0.86	0.46	0.80	NA
Folic acid (μg/100 g)	ND	0.55	20.14	0.28	NA
Biotin (μg/100 g)	ND	246.40	7.03	ND	NA
**Amino acids**					
Aspartic acid (g/100 g)	0.40	0.59	0.64	0.81	NA
Threonine (g/100 g)	0.22	0.27	0.43	0.36	NA
Serine (g/100 g)	0.26	0.23	0.33	0.30	NA
Glutamic acid (g/100 g)	0.97	1.00	1.11	1.39	NA
Proline (g/100 g)	0.49	0.33	0.49	0.47	NA
Glycine (g/100 g)	0.12	0.11	0.13	0.16	NA
Alanine (g/100 g)	0.20	0.27	0.31	0.36	NA
Valine (g/100 g)	0.36	0.34	0.40	0.48	NA
Methionine (g/100 g)	0.15	0.15	0.15	0.21	NA
Isoleucine (g/100 g)	0.30	0.34	0.40	0.48	NA
Aminocaproic acid (g/100 g)	0.52	0.69	0.69	0.95	NA
Tyrosine (g/100 g)	0.20	0.21	0.21	0.29	NA
Phenylalanine (g/100 g)	0.25	0.26	0.23	0.36	NA
Lysine (g/100 g)	0.46	0.54	0.62	0.76	NA
Histidine (g/100 g)	0.16	0.13	0.14	0.19	NA
Arginine (g/100 g)	0.21	0.18	0.18	0.25	NA
Cystine (g/100 g)	0.11	0.28	0.17	0.35	NA
Tryptophan (g/100 g)	0.04	0.06	0.11	0.08	NA

^(a)^ Milk TC refers to the Tsang and Collins formula [40]. ^(b–d)^ Milk A (possum milk substitute < 0.8) and milk C (possum milk substitute > 0.8) are milk substitutes for possums; milk B is a milk substitute for guinea pigs. The three milk substitutes are commercially available from Wombaroo Food Products of Australia. ^(e)^ Values were taken from Yang et al. [39]. ^(f)^ NA, not available. ^(g)^ ND, not detectable.

**Table 2:** Body weight of the Chinese tree shrew pups fed with different milks.

Age	Maternal Milk (MR)		Milk TC		Milk A		Milk B		Milk C	
	No. of Pups	Weight (g)	No. of Pups	Weight (g)	No. of Pups	Weight (g)	No. of Pups	Weight (g)	No. of Pups	Weight (g)
Postnatal day 1 ^(a)^	21	10.64 ± 0.32	20	10.57 ± 0.23	8	10.03 ± 0.47	67	10.46 ± 0.14	17	9.41 ± 0.23 #,†,¥
Postnatal day 5	222	15.62 ± 0.20	11	12.43 ± 0.53 #	12	14.05 ± 0.42	213	14.81 ± 0.14 †	34	13.49 ± 0.40 #,¥
Postnatal day 10	213	25.16 ± 0.45	1	13.40	6	20.14 ± 0.52	202	22.28 ± 0.30 #	29	20.40 ± 0.77 #
Postnatal day 20	194	47.08 ± 0.83	0	—	3	36.90 ± 0.78 #	184	42.41 ± 0.57 #,‡	21	39.67 ± 2.04 #
Postnatal day 30	183	63.60 ± 1.08	0	—	0	—	182	56.60 ± 0.66 #	19	60.45 ± 2.02
Postnatal day 40	180	81.08 ± 1.05	0	—	0	—	182	72.54 ± 0.74 #	19	74.06 ± 2.24 #
Postnatal day 50	178	91.90 ± 0.80	0	—	0	—	182	85.24 ± 0.80 #	19	90.12 ± 2.28
Postnatal day 60	175	102.8 ± 0.74	0	—	0	—	181	98.10 ± 0.72 #	18	100.4 ± 1.77

Differences in body weight among groups were analyzed by ANOVA, followed by post-hoc Bonferroni test. Welch ANOVA and post-hoc Tamhane’s T2 tests were used if the assumption of homogeneity of variance was violated. When the Shapiro–Wilk test for Gaussian distribution failed, the non-parametric Kruskal–Wallis test and post-hoc Dunn test were performed. ^(a)^ On postnatal day 1, several pups (maternal milk, *n* = 203; milk TC, *n* = 2; milk A, *n* = 4; milk B, *n* = 147; and milk C, *n* = 18) that sucked colostrum immediately after birth were not measured for body weight data and were excluded from the analyses. #, a significant difference (*p* < 0.05) was observed between the MR group and each of the four sub-groups (milk TC, milk A, milk B, or milk C) in the AR group. †, a significant difference (*p* < 0.05) was observed between pups fed with milk TC and each of the other three milks (milk A, milk B, or milk C) in the AR group. ‡, a significant difference (*p* < 0.05) was observed between pups fed with milk A and milk B, and between milk A and milk C. ¥, a significant difference (*p* < 0.05) was observed between pups fed with milk B and milk C.

**Table 3:** Number of deaths before the weaning of tree shrew pups and survival rate at the weaning of pups fed with different milks.

Milk	No. of Pups	No. of Deaths (Percentage)				Survival Rate at the Weaning Age (%) ^(a)^
		Respiratory Diseases	Digestive Diseases	Other or Unknown Diseases	Sub-Total	
Maternal milk (MR)	224	8 (17.8%)	23 (51.1%)	14 (31.1)	45	79.9%
Milk TC	22	2 (9.1%)	20 (90.9 %)	0 (0%)	22	0.0% #
Milk A	12	3 (25.0%)	5 (41.7%)	4 (33.3%)	12	0.0% #
Milk B	214	7 (21.9%)	15 (46.9%)	10 (31.3%)	32	85.0% †,‡
Milk C	35	5 (31.3%)	7 (43.8%)	4 (25.0%)	16	54.3% #,†,‡,¥

^(a)^ Differences of the survival rate at the weaning age between two groups were analyzed by the Fisher’s exact test. #, a significant difference (*p* < 0.05) was observed between the MR group and each of the four sub-groups (milk TC, milk A, milk B, or milk C) in the AR group. †, a significant difference (*p* < 0.05) was observed between pups fed with milk TC and each of the other three milks (milk A, milk B, or milk C) in the AR group. ‡, a significant difference (*p* < 0.05) was observed between pups fed with milk A and milk B, and between milk A and milk C. ¥, a significant difference (*p* < 0.05) was observed between pups fed with milk B and milk C.

**Table 4:** Comparison of the life span and reproductive performance of the F_1_ generation tree shrews fed with maternal milk and milk B in the inbreeding colonies during 2013–2021.

Item	Sex	Maternal Milk		Milk B		*p* ^(b)^
		No. of Animals	Value	No. of Animals	Value	
Life span (years old) ^(^^a)^	male	279	4.07 ± 0.14	57	4.36 ± 0.22	0.712
	female	265	4.19 ± 0.14	53	3.81 ± 0.25	0.217
Age at the first reproduction (years old)	male	138	1.62 ± 0.08	40	1.19 ± 0.08	0.053
	female	170	1.61 ± 0.07	47	1.03 ± 0.05	<0.001
Litter size ^(c)^	male	138	2.94 ± 0.07	40	2.95 ± 0.07	0.885
	female	170	2.95 ± 0.09	47	2.98 ± 0.07	0.714
Number of litters ^(d)^	male	138	7.91 ± 0.62	40	9.70 ± 1.07	0.058
	female	170	5.56 ± 0.34	47	8.38 ± 0.60	<0.001
Survival rate of the offspring at the weaning age (%) ^(e)^	female	170	74.1 (294/397)	47	85.8 (357/416)	<0.001

^(a)^ Data of life span were collected for the F_1_ generation tree shrews with an age older than 60 days but died before the end of 2021. ^(b)^ Differences of each parameter (including life span, age at the first reproduction, litter size and number of litters) between tree shrews fed with maternal milk and milk B were analyzed by the Mann–Whitney U test, while the survival rates of offspring at the weaning age between female tree shrews fed with maternal milk and milk B were compared by the Fisher’s exact test. ^(c)^ Litter size was defined as the number of offspring produced at one birth by a parent tree shrew. ^(d)^ The number of litters was determined by counting the total number of litters of offspring produced by a parent tree shrew, and individuals that produced no offspring were excluded from this analysis. ^(e)^ The survival rate of the offspring at the weaning age was measured by the ratio of the offspring survived to the weaning age to the number of total offspring with exclusion of those born alive but died of infanticide.

## Data Availability

The data presented in this study are available on request from the corresponding author.
